# A 10-week implementation of the FIT FIRST FOR ALL school-based physical activity concept effectively improves cardiorespiratory fitness and body composition in 7–16-year-old schoolchildren

**DOI:** 10.3389/fpubh.2024.1419824

**Published:** 2024-07-17

**Authors:** Helgi Winther Olsen, Tórur Sjúrðarson, Bára Berghamar Danielsen, Peter Krustrup, Malte Nejst Larsen, May-Britt Skoradal, Magni Mohr

**Affiliations:** ^1^Faculty of Education, University of the Faroe Islands, Tórshavn, Faroe Islands; ^2^Center of Health Science, Faculty of Health, University of the Faroe Islands, Tórshavn, Faroe Islands; ^3^Department of Sports Science and Clinical Biomechanics, SDU Sport and Health Sciences Cluster (SHSC), Faculty of Health Sciences, University of Southern Denmark, Odense, Denmark; ^4^Danish Institute for Advanced Study (DIAS), University of Southern Denmark, Odense, Denmark

**Keywords:** health-related fitness, health status, cardiorespiratory fitness, body composition, agility, physical activity concept, school-based, FIT FIRST

## Abstract

**Introduction:**

The purpose of the present study was to investigate the impact of the FIT FIRST FOR ALL school-based physical activity program on health-related physical fitness in Faroese schoolchildren. The program aimed to add three weekly sessions of organized high-intensity physical activity to the standard weekly physical education sessions for all pupils across the entire school.

**Methods:**

A non-randomized controlled design was used to evaluate the effects of the program. Two schools participated, including one intervention school (INT; *n* =179) and one control school (CON; *n* =181), with pupils aged 7–16 years (grades 1–9). The FIT FIRST FOR ALL program consisted of three weekly 40-minute sessions of age-adjusted high-intensity physical activity over 10 weeks for the INT school, while the CON school continued their normal school program. Pre- and post-intervention assessments included cardiorespiratory fitness (Yo-Yo IR1C test), agility (Arrowhead Agility test), postural balance (Stork Stand), standing long jump performance, body composition, blood pressure, and resting heart rate.

**Results:**

A significant time × group effect (*p* < 0.001) was observed for cardiorespiratory fitness, which increased by 31% [23;39] in INT (*p* < 0.001) and remained unaltered in CON (7% [−2;16], *p* = 0.13). In addition, a time × group effect (*p* < 0.001) was observed for agility, which improved by 2.1% [1.0;3.2] in INT (*p* < 0.001) and regressed by 3.3% [2.3;4.4] in CON (*p* < 0.001). No significant between-group effects were found for standing long jump and balance. A time × group effect (*p* < 0.001) was observed for changes in total muscle mass, which increased by 1.4 kg [1.2;1.5] in INT (*p* < 0.001) and by 0.4 kg [0.3;0.6] in CON (*p* < 0.05). Furthermore, a time × group effect (*p* < 0.001) was observed for total fat percentage, which decreased by −2.3% [−2.8;−1.9] in INT (*p* < 0.001) and remained unchanged in CON (−0.3% [−0.7;0.1], *p* = 0.16). No significant time × group effects were found for blood pressure and resting heart rate.

**Discussion:**

The FIT FIRST FOR ALL program significantly improved cardiorespiratory fitness and agility, and it led to favorable changes in body composition in the intervention school. These findings suggest that the program is highly effective in enhancing physical fitness and health status across all investigated age groups when implemented at a school-wide level.

## 1 Introduction

Global physical activity levels have declined while sedentary behavior has risen over recent decades with one in three adults failing to meet the minimum requirements of physical activity (1, 2), which is a major health concern (3–5). Furthermore, only a fourth to a third of children and adolescents worldwide meet the recommended 60 min of moderate to vigorous physical activity per day (6). Thus, sustainable, and efficient physical activity models are highly warranted, especially for children and adolescents.

Several studies have demonstrated a strong correlation between physical activity (7) or sport participation (8, 9) and both physical and mental health status among children and adolescents. For example, regular participation in sporting activities, particularly team sports, has been shown to markedly improve cardiovascular and musculoskeletal health as well as general well-being (10).

Schools may present an ideal setting for implementing physical activity interventions since most children and adolescents spend a substantial part of their weekly hours in school. Moreover, the presence of a social gradient in health and fitness (11, 12), potential gender differences (13–16) and differences in leisure time sports-participation among children of diverse ethnicities (17) can be effectively addressed through school-based models targeting all children irrespective of demographics. However, despite the potential, a recent review (18) on the health effects of school-based physical activity interventions concludes that many of the investigated interventions lead to either minimal or small effects, hence further investigation into new interventions remains crucial.

Physical education is mandatory in Faroese schools, as well as in other Scandinavian countries through grade 1 to 9 and follows a national curriculum that includes equal parts of swimming, ballgames, gymnastics, dance, athletics, and outdoor activities. In grade 1 pupils are provided 30 h a year of PE, and for each year in grade 2 through 9 they are given 60 h yearly. This corresponds to approximately 1–2 h a week. PE teaching is mainly delivered by trained PE teachers and is focused on introducing pupils to and training them in a variety of physical and technical sports related activities. While PE is mandatory, exams or physical assessments are not mandated, although pupils are evaluated by the national grading system. A 2022 national questionnaire assessed the level of participation in organized leisure time sports for Faroese children and adolescents to 71% for 9–15-year-olds and 52% for 16–18-year-olds, whereof 31% being active once or twice a week or less, 35% being active 3–4 times a week, and 34% participating more than 4 times a week (19). The self-reported data have not been validated by objectively measured physical activity, and with self-reported data often being higher than objectively measured values, it seems plausible to conclude that many Faroese children do not meet the recommended daily 60 min on a weekly basis. A 2024 report shows that obesity among 7–8-year-old Faroese children is between the highest levels in Europe with 15.0% of being overweight (CDC 85–94th percentile), 11.2% obese (CDC 95–99th percentile) and 4.1% severely obese (CDC ≥99th percentile) (20, 21). Thus, the global pandemic of physical inactivity and obesity in children also appears to be a challenge in small-scale island societies such as the Faroe Islands.

Taken together, with the Faroese society experiencing challenges with sedentarism and lack of active transport to school combined with the challenges of increased screen time and poor dietary habits, the importance of organized campaigns striving to champion healthier habits for children is increasingly important.

A new school-based physical activity concept known as FIT FIRST (22) comprises three 40 min weekly sessions for the duration of a school year and is intended to supplement curricular physical education teaching with a ready-to-use physical activity plan. Each session consists of a 40 min activity plan featuring inclusive, fun, and high-intensity games inspired from more than 20 different sports ranging from ball games to athletics and martial arts. The program includes detailed plans for four to six sessions in each thematic sport with more sessions being developed as the program is continuously being advanced. It is meticulously designed to enhance the pupils’ cardiovascular fitness, muscle strength and bone tissue density through a high intensity training load with high heart rate, anaerobic energy turnover as well as maximal muscle contractions while impacting bone by mechanical strain and ground reaction forces. The efficiency of the program to elicit the intended physical load has been validated throughout the development phase using accelerometer and heart rate measurements to include sessions that elicited high degrees of time in motion and high average heart rate response above 75% and preferably over 80% of HRmax and with 10% of the time spent over 90% HRmax (23, 24).

The FIT FIRST program originates in Denmark and was developed in collaboration between researchers from the University of Southern Denmark, the Danish Sports Confederation and Team Denmark (22, 23). There are three age-related FIT FIRST programs designed for each of the grades 1–3 (7–9 years), grades 4–6 (10–12 years) and grades 7–9 (13–16 years) in the Danish primary and lower secondary school. Together these programs define the FIT FIRST FOR ALL. Most schools are satisfied with the program and would recommend others to implement it, but many mention the facilities at their school as a barrier for many classes using the program at the same time (25). To date, the FIT FIRST program has not been tested in an entire school, encompassing children aged 7–16 years. Therefore, the purpose of the present study is to assess the effect of FIT FIRST on physical health when it is implemented school-wide over a 10-week period with three weekly sessions.

We hypothesize that the FIT FIRST FOR ALL program can improve our primary endpoints (cardiorespiratory fitness and body composition), as well as our secondary endpoints (agility, balance, jump performance, resting heart rate and blood pressure) when implemented at a school-wide level. As an explorative hypothesis, we test that these effects are gender and age dependent.

## 2 Methods

### 2.1 Study design

We applied a non-randomized controlled trial design where two schools on the Faroe Islands were assigned into an intervention school and a control school. The 10-week intervention period was carried out during the second half of the school year from February through to May with tests being carried out before and after the intervention period. The schools are geographically located on the same island in towns having a similar size considering the regional differences on the Faroe Island (26). Also, the two schools have the same size (around 200 pupils in each school), and the schools are comparable in relation to gender distribution in each age group. As per definition in the Faroe Islands school system the grades 1 to 9 are divided into three subdivisions with grades 1–3 as level I (7–9 years), grade 4–6 as level II (10–12 years) and grades 7–9 as level III (13–16 years). To investigate possible differences in age groups the same subdivisions were used for data treatment.

### 2.2 Participants

Pupils attending grade 1 to grade 9, aged 7–16 years, were invited to participate in the research project, both in the intervention school (INT, *n* = 190) and the control school (CON, *n* = 191). All students in classes with special needs (INT, *n* = 14, CON, *n* = 14) and pupils from grade 10 (INT, *n* = 25, CON, *n* = 12) were excluded from the study. Of a total of 381 eligible pupils, the parents of 365 pupils gave written consent for participation. By their own choice 4 pupils did not wish to participate in the testing, which resulted in a total of 361 pupils participating (INT, *n* = 179, CON, *n* = 181). Table 1 shows the baseline characteristics of the level I, II and III pupils.

**Table 1:** Participant characteristics.

Variable	Level	Intervention		Control		*p*-value
		Mean ± SD	*N*	Mean ± SD	*N*	
Age (yrs.)	I	8.8 ± 0.9	65	8.7 ± 0.9	56	0.54
	II	11.8 ± 0.9	59	11.8 ± 0.9	68	0.85
	III	14.7 ± 1.0	55	14,6 ± 0.9	57	0.58
	ALL	11.6 ± 2.6	179	11.7 ± 2.5	181	0.68
Height (cm)	I	134 ± 8	65	134 ± 8	56	0.99
	II	155 ± 9	59	155 ± 10	68	0.66
	III	168 ± 8	55	166 ± 8	57	0.15
	ALL	152 ± 16	179	152 ± 16	181	0.81
Weight (kg)	I	34.2 ± 9.6	65	31.9 ± 7.9	56	0.17
	II	50.4 ± 13.3	59	50.1 ± 11.8	68	0.89
	III	64.6 ± 14.0	55	65.5 ± 17.9	57	0.77
	ALL	49.0 ± 17.5	179	49.1 ± 18.7	181	0.95
BMI	I	18.7 ± 3.4	65	17.5 ± 2.7	56	0.04
	II	20.9 ± 4.3	59	20.6 ± 3.6	68	0.70
	III	22.7 ± 4.2	55	23.7 ± 5.5	57	0.33
	ALL	20.7 ± 4.3	179	20.6 ± 4.7	181	0.82
BMIz	I	0.76 ± 0.88	65	0.37 ± 0.94	56	0.03
	II	0.56 ± 1.20	59	0.61 ± 1.05	68	0.84
	III	0.61 ± 0.95	55	0.81 ± 0.94	57	0.29
	ALL	0.65 ± 1.02	179	0.59 ± 0.99	181	0.62
Total fat (%)	I	25.7 ± 8.4	65	23.3 ± 7.9	56	0.12
	II	26.8 ± 10.5	59	25.5 ± 10.2	68	0.53
	III	24.7 ± 10.4	55	25.5 ± 10.7	57	0.69
	ALL	25.8 ± 9.7	179	24.8 ± 9.7	181	0.38
Muscle mass (kg)	I	12.6 ± 2.9	65	12.3 ± 2.8	56	0.53
	II	19.3 ± 4.0	59	19.8 ± 4.6	68	0.49
	III	26.4 ± 5.1	55	26.5 ± 5.9	57	0.89
	ALL	19.1 ± 6.9	179	19.5 ± 7.3	181	0.57
MAP (mmHg)	I	74 ± 7	65	72 ± 8	56	0.30
	II	75 ± 7	59	76 ± 7	68	0.51
	III	81 ± 8	55	83 ± 10	57	0.25
	All	76 ± 8	179	77 ± 9	181	0.46
Gender distribution (M/F (%))	I	(52/48)	65	(41/59)	56	0.22
	II	(51/49)	59	(60/40)	68	0.29
	III	(45/55)	55	(53/47)	57	0.45
	ALL	(50/50)	179	(52/48)	181	0.71

Continuous variables are presented as means ± standard deviations, and dichotomous data as numbers (percentages). BMI, body mass index; BMIz, body mass index z-score; MAP, mean arterial pressure. Continuous data are compared using an unpaired *t*-test, and proportions using a Pearson’s chi squared test.

### 2.3 FIT FIRST physical activity sessions

Four physical education (PE) teachers in the intervention school were trained to deliver the FIT FIRST sessions during a two-day course a month prior to the intervention and were given support to plan a 30-session FIT FIRST program for the duration of 10 weeks with 3 sessions per week. The respective age-related versions of FIT FIRST FOR ALL (23) were used for levels I (FIT FIRST 10), II (FIT FIRST 20) and III (FIT FIRST Teen) in the intervention school and the PE teachers were instructed to construct a lesson plan using a variety of the program while using the same thematic sport for a week at a time. They were free to select between the thematic sports they felt most inspired to teach in. The 3 times 40 min weekly FIT FIRST physical activity sessions were given in addition to the mandatory 60–120 min weekly physical education.

To streamline and optimize the time pupils were active during the FIT FIRST sessions, class teachers assisted in quickly transferring pupils between classroom teaching and FIT FIRST sessions held in the school PE facility or outdoors and how to support the trained PE teachers during FIT FIRST sessions. A record of completed sessions and pupil attendance was kept during the intervention period. From the planned 3 weekly sessions an average of 2.3 ± 0.2, 2.2 ± 0.0 and 2.1 ± 0.1 sessions per week, respectively, were completed for levels I, II and III. The pupil attendance was 84 ± 11, 87 ± 11 and 75 ± 24%, respectively, for levels I, II and III during the completed sessions.

### 2.4 Test protocols

Pre- and posttests were carried out over the span of 2 to 3 days before and after the intervention. On separate days all pupils went through two test batteries consisting of (1) body composition and blood pressure measurements carried out in dedicated classrooms and (2) exercise capacity tests consisting of the Yo-Yo Intermittent Recovery level 1 Children’s test (YYIR1C), a horizontal Standing Long Jump test (SLJ), and an Arrowhead Agility test (AAT), as well as a Stork Stand test for postural balance (BAL) carried out in an indoor sports hall. Care was taken that blood pressure measurements preceded the exercise capacity tests or any other physical activity.

### 2.5 Body composition

Body composition was measured with an InBody 270 multi-frequency body composition analyzer (Biospace, California, United States) as previously described (27). The recorded outcome was body weight, body fat percentage and total muscle mass. Height was measured with an analogue Seca 217 stadiometer (Seca, Hamburg, Germany). The approach was recently tested against measurements obtained with dual-energy X-ray absorptiometry, which showed excellent inter-device absolute and relative reliability, but an underestimation of muscle mass and overestimation of body fat percentage (27). An estimate of the initial degree of overweight in the test population was derived from body mass index-for-age z-scores (BMIz) calculated with the CDC Anthropometry R-package with extended scores for children with obesity (28). Category cut-offs were defined by standard deviations as thinness ≤ −2 SD, normal range −2 to 0.99 SD, overweight 1 to 1.99 SD and obesity ≥2 SD (29). All changes in body composition were estimated based on the direct measurements of body fat percentage and total muscle mass, as BMIz has been shown to be less sensitive to track changes in body composition (30, 31).

### 2.6 Blood pressure and resting heart rate

Blood pressure was measured once on both the right and the left arm and the side with highest values was chosen for two additional measurements. Measurements were carried out with an automatic blood pressure monitor (Omron, M3, HEM-7154-E) in a separate and quiet room where pupils were asked to sit quietly on a chair with their arms relaxing on the table (32). The procedure was simplified for the youngest pupils aged 7–9 years old, where blood pressure was measured three times on the left side only because of difficulties with cuff fit to small arm sizes and the ability of the children to focus for the required time. Mean arterial blood pressure was calculated as the means from the final two measurements. Resting heart rate was also determined during the blood pressure measurements, as previously described (15).

### 2.7 Yo-Yo Intermitten Recovery 1 Children’s test

The Yo-Yo Intermittent Recovery 1 Children’s Test is an audio supported shuttle run test. At the cue of a taped voice, shuttle runs were performed at increasing speed between two lines separated by 16 m, followed by a 10 s recovery jog around a marker placed 4 m behind the starting line (33). Two instructors were guiding pupils at the start line and an instructor was running together with the youngest pupils aged 7–9 years. for the entire duration of the test to support the audio cues with visual pacing. Two investigators were noting performance outcomes with the support of video recordings as backup. All pupils were encouraged to run until exhaustion. If pupils during the test did not meet the time demands of a shuttle they were encouraged to hurry for the next shuttle. When a pupil failed to meet the time demands twice or otherwise stopped, the total distance completed including the final shuttle was recorded as the test result for analysis.

Heart rate was measured during the YYIR1C test with Polar Team Pro (Polar Electro Oy, Kempele, Finland) HR monitor system to investigate peak heart rate over 15 s (HR_peak_) and validate level of exhaustion (34).

### 2.8 Arrowhead Agility test

From a standing sprint position with one leg in front at a starting line, pupils were instructed to run twice in an arrow shaped pattern as fast as possible. Once to the right and then once to the left. The arrow shape was marked by cones at 5 m and 10 m in a straight line from the starting line and at 5 m to the side of the 5 m cone. If pupils touched the cones or missed the intended pattern, they were asked to retry. Time was measured with photocells and an electronic timing system (Babaly Timing, Wireless Sport Timing System, Hungary) at the starting line and the combined time for both runs was used as the result for analysis (35).

### 2.9 Standing Long Jump test

From a standing position with arms held over the head and toes on both feet at a starting line, pupils were asked to swing their arms in one motion down and back while bending their knees, and on the forward swing to jump as far as possible to land on two feet. Jump length was measured from the start line to the rear of the backmost heel with a measuring tape. Pupils were instructed before their jumps and were allowed to practice a few times before testing. Failed jumps were allowed a repeat and the longest of two successful test jumps was recorded as the result for analysis (36).

### 2.10 Stork Stand Balance test

Pupils were asked to stand on the floor and place their hands on their hips, lift the left foot and place it against the right knee. The test would start when the heel of the standing foot was raised from the floor and students would hold the position for as long as possible. The test would end if the foot lost contact with the knee, hands left the side, or the supporting foot moved or touched the floor. Three attempts were carried out on each foot, first right and then left, with short voluntary breaks between attempts and time was recorded for each attempt. The longest measured attempt on each leg was recorded as the result for analysis (37).

### 2.11 Ethics perspectives

The research project was approved by the Research Ethics Council of the Faroe Islands. Teachers and staff of both schools were informed about the methods used and were given an active voice in the planning of the procedures. All pupils were under 18-year-olds and thus by Faroese law defined as minors. Parents were informed by sending an information letter 7 days prior to collective information meetings at each school, and written consent was obtained from all parents or guardians. All pupils were also informed about the research project by their parents and their head-teacher and were informed by the researchers on the days of data collection. The FIT FIRST physical activity sessions at the intervention school were mandatory but every pupil was informed that research participation was voluntary even though their parents had consented. All data collected was anonymized guaranteeing total anonymity for each participant according to Faroese Data Protection law.

### 2.12 Missing data

Data from students with informed consent collected at pre- and posttest was included. A track record of absence from the mandatory FIT FIRST lessons was kept at the intervention school but all pupils regardless of adherence were included.

### 2.13 Statistical analyses

**Figure 1:**
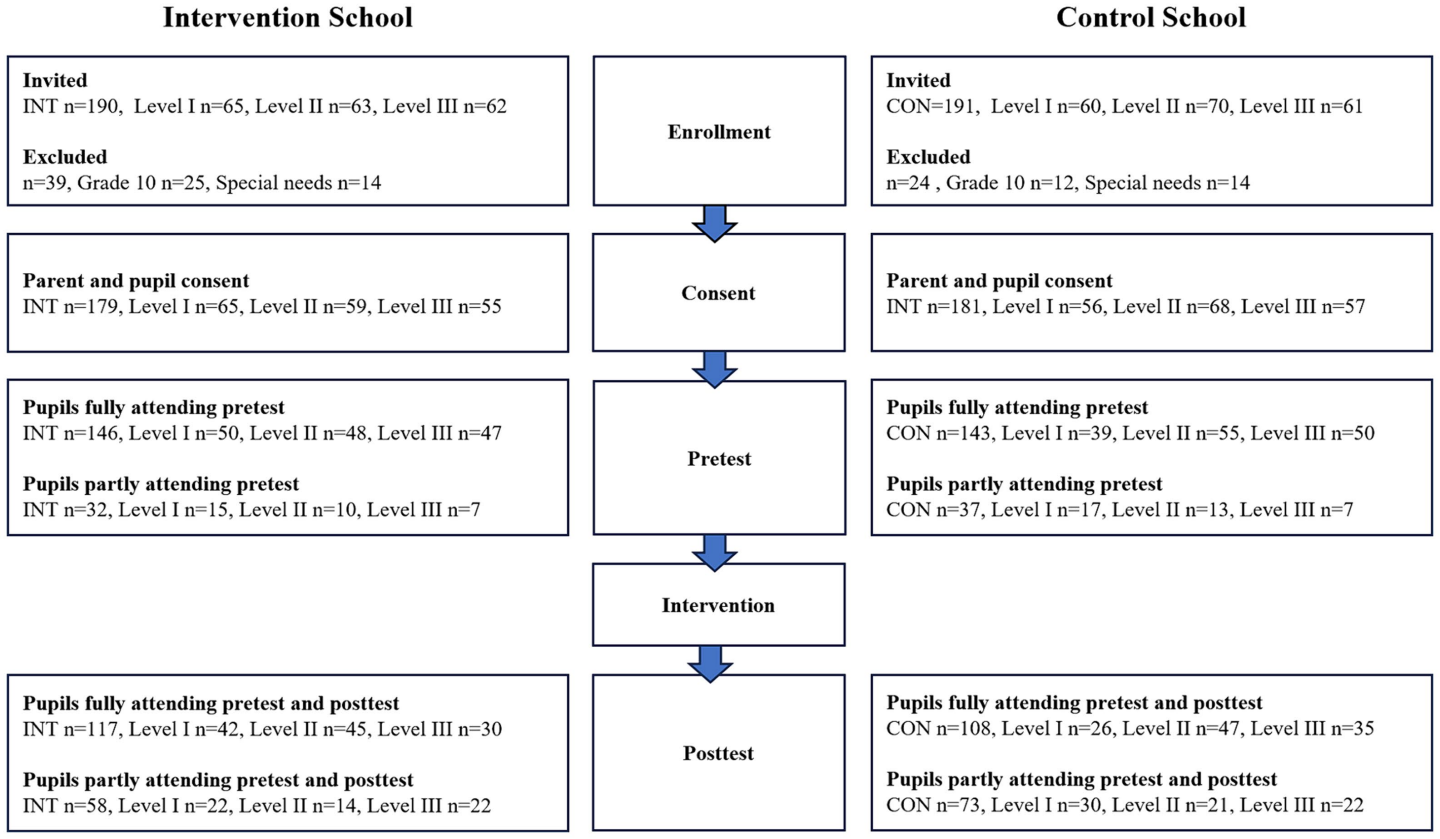
Schematic flow chart of pupil participation.

Data are presented as means with 95% confidence intervals. The statistical analysis was conducted based on the intention-to-treat principle, which included all randomized participants. Continuous endpoints were analyzed by a linear mixed model for repeated measures approach using SPSS (IBM SPSS Statistics, version 28.0.0) (38). The primary analysis included time (pre vs. post), group (intervention vs. control), and a time × group interaction as fixed effects. The interaction term was used to assess whether the two groups (intervention school and control school) demonstrated different responses in the obtained outcomes over time. A Sidak-adjusted pairwise comparison was completed for all endpoints. Random variation and repeated effects were defined from participants. Independence of the obtained data was assumed in the model. All data were subjected to visual inspection of normality of the residuals and homogeneity of residual variance. Furthermore, an independent samples *t*-test was used to assess potential between-gender (boys vs. girls) differences in the relative changes (%) from baseline in selected outcomes to assess the influence of gender. Finally, a one-way ANOVA was also conducted to evaluate possible between-level differences (level I vs. level II vs. level III) in the relative changes (%) from baseline in selected outcomes to assess the impact of age. The level of statistical significance was set at *p* < 0.05 (Figure 1).

## 3 Results

### 3.1 Exercise capacity

**Figure 2:**
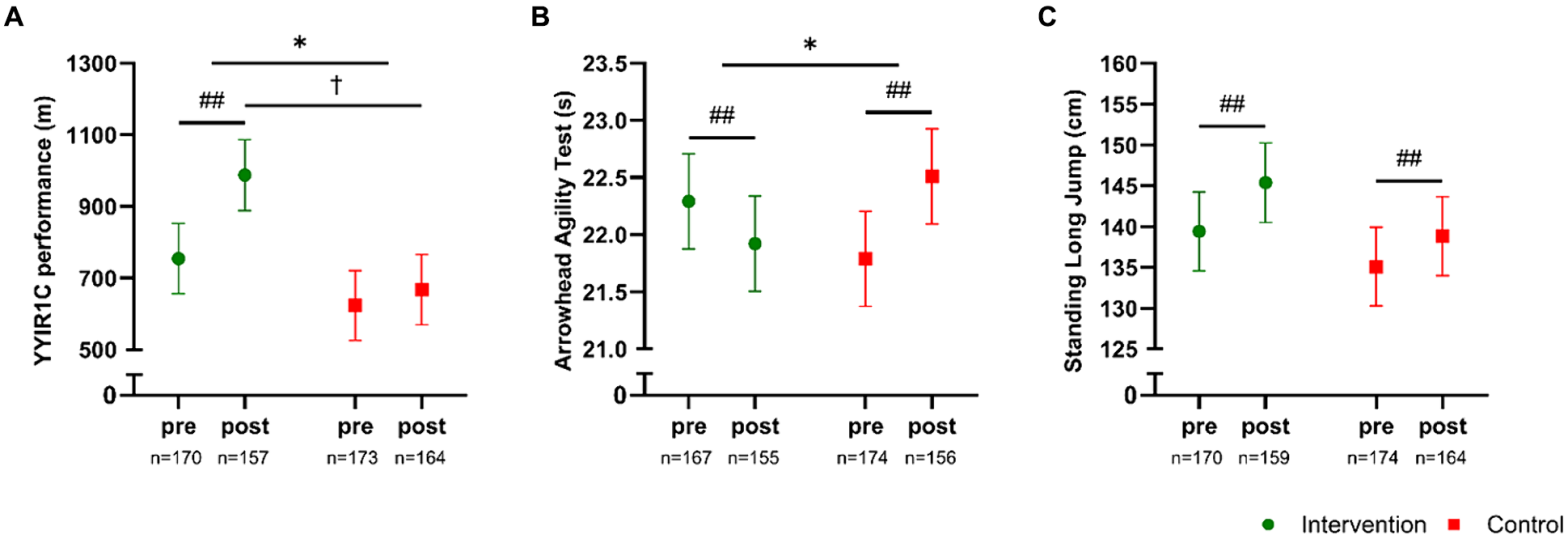
Yo-Yo Intermittent Recovery 1 Children’s test **(A)**, Arrowhead Agility test **(B)**, and Standing Long Jump test **(C)** measured pre- and post-intervention in participants in the intervention school (green) and the control school (red). Values are presented as means with 95% confidence intervals from a linear mixed model with repeated measures including time, group and time × group as fixed effects. * Denotes a “time × group” interaction at *p* < 0.05. The result of the post hoc analysis is indicated by ## *p* < 0.001 compared with baseline, and †*p* < 0.05 compared with control.

A significant time × group effect (*p* < 0.001) existed for changes in cardiorespiratory fitness in favor of the intervention group. Specifically, a significant (*p* < 0.001) increase in YYIR1C performance of 233 m [174;292] was observed in INT, whereas a non-significant (*p* = 0.13) numeric increase of 45 m [−13;102] existed in CON, resulting in a 320 m higher (*p* < 0.001) YYIR1C performance [181;459] in INT compared to CON post-intervention (Figure 2A). In addition, a significant time × group effect (*p* < 0.001) was observed for changes in agility in favor of the intervention group. Specifically, INT improved their time to complete the agility test “arrowhead” by −0.5 s [−0.7;−0.2], whereas CON demonstrated a worsened (*p* < 0.001) time by 0.7 s [0.5;1.0] (Figure 2B). Furthermore, the standing long jump increased (*p* < 0.001) in both groups, with magnitudes of 6.0 cm [3.7;8.3] in INT and 3.7 cm [1.5;5.9] in CON, but with no statistical difference between groups (time × group, *p* = 0.16) (Figure 2C). Finally, no between-group effect (*p* = 0.97) existed for changes in postural balance.

### 3.2 Body composition

**Figure 3:**
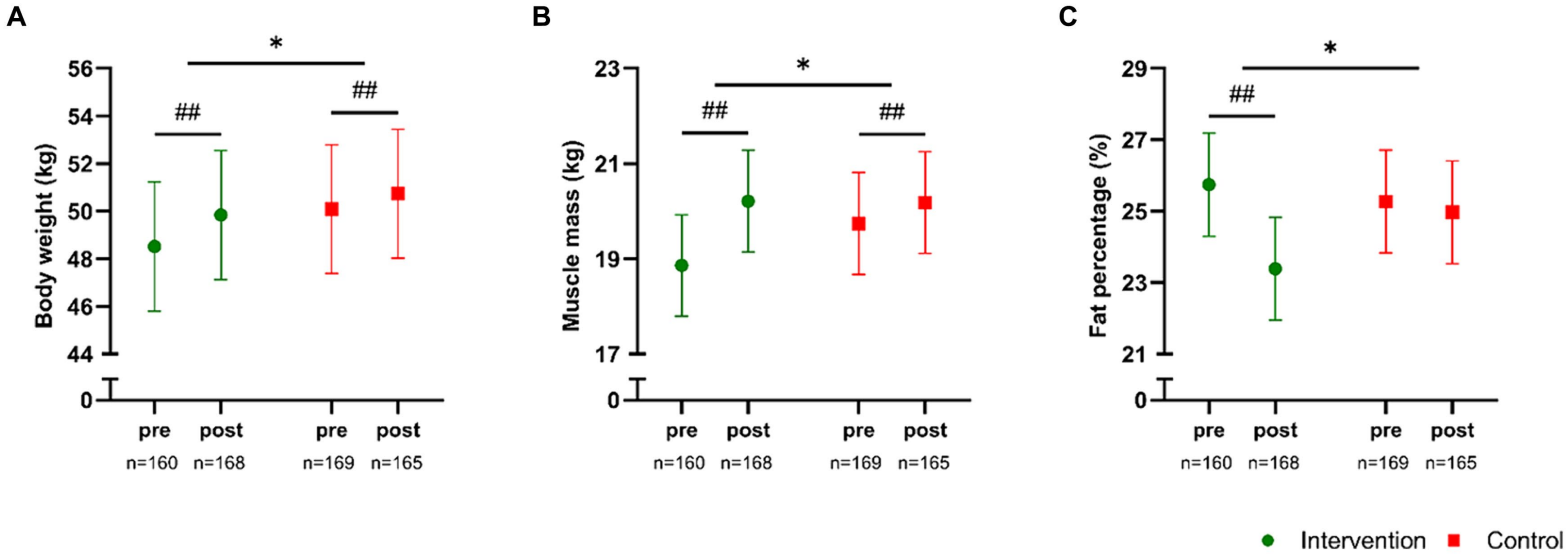
Body weight **(A)**, total muscle mass **(B)**, and total fat percentage **(C)** measured pre- and post-intervention in participants in the intervention school (green) and the control school (red). Values are presented as means with 95% confidence intervals from a linear mixed model with repeated measures including time, group and time × group as fixed effects. * Denotes a “time × group” interaction at *p* < 0.05. The result of the *post hoc* analysis is indicated by ##*p* < 0.001 compared with baseline.

A significant between-group effect (*p* < 0.001) existed for total body weight, which increased (*p* < 0.001) in both groups, with magnitudes of 1.3 kg [1.1;1.6] in INT and 0.6 kg [0.4;0.9] in CON (Figure 3A), and this between-group effect in body weight may have been related to a larger increase in height (time × group, *p* < 0.001) in INT (2.5 cm [2.3;2.7], *p* < 0.001) compared to CON (1.4 cm [1.2;−1.6], *p* < 0.001). In addition, a time × group effect (*p* < 0.001) was observed for changes in total muscle mass, which increased (*p* < 0.001) by 1.4 kg [1.2;1.5] in INT and by 0.4 kg [0.3;0.6] in CON (Figure 3B). Furthermore, a significant time × group effect (*p* < 0.001) existed for total fat percentage, which decreased (*p* < 0.001) by −2.3% [−2.8;−1.9] in the INT group and remained unchanged in CON (−0.3% [−0.7;0.1], *p* = 0.16) (Figure 3C).

### 3.3 Blood pressure and resting heart rate

**Figure 4:**
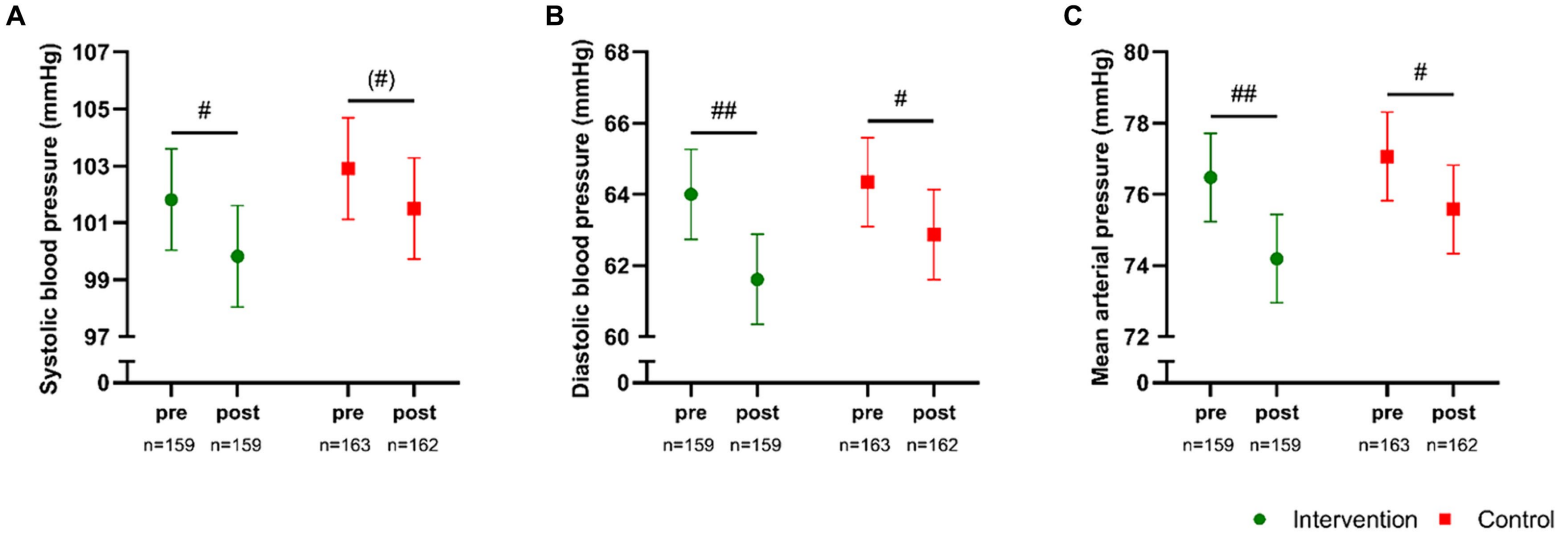
Systolic blood pressure **(A)**, diastolic blood pressure **(B)**, and mean arterial pressure **(C)** measured pre- and post-intervention in participants in the intervention school (green) and the control school (red). Values are presented as means with 95% confidence intervals from a linear mixed model with repeated measures including time, group and time × group as fixed effects. * Denotes a “time × group” interaction at *p* < 0.05. The result of the *post hoc* analysis is indicated by (#)*p* = 0.05, #*p* < 0.05, ##*p* < 0.001 compared with baseline.

No significant between-group effect (*p* ≥ 0.34) existed for SBP, DPB or MAP (Figures 4A–C). However, the *post hoc* analysis revealed a significant reduction in SBP of −2.0 mmHg [−3.4;−0.5, *p* = 0.007] and in DBP of −2.4 mmHg [−3.7;−1.0, *p* < 0.001], resulting in a reduction (*p* < 0.001) in MAP of −2.3 mmHg [−3.4;−1.1] in INT. Similar observations were demonstrated in the CON group, with a borderline significant (*p* = 0.05) reduction in SBP of −1.4 mmHg [−2.8;0.0] and a significant (*p* = 0.03) reduction in DBP of −1.5 mmHg [−2.8;−0.1], resulting in a reduction (*p* = 0.01) in MAP of −1.5 mmHg [−2.6;−0.3]. No between-group or within-group effect existed for resting heart rate.

### 3.4 Sub-group analysis – the effect of gender

In general, the intervention-induced changes in the obtained variables were similar between both genders (Table 2). However, the improvements in body composition induced by the intervention were influenced by gender. Relative (%) to baseline measurements, muscle mass increased (*p* < 0.001) by 8.2% [7.0;9.5] (from 19.2 kg [17.7;20.6] to 20.7 kg [19.3;22.2]) for boys, which was more pronounced (*p* < 0.05) than the increase of 6.1% [4.9;7.4] (from 18.6 kg [17.1;20.0] to 19.7 kg [18.2;21.2]) observed for girls. Moreover, the marked reduction in total fat percentage of −12.8% [−15.5;−10.2] (from 22.5% [20.6;24.5] to 19.6% [17.7;21.6]) among the boys, was superior (*p* < 0.001) to the reduction of −6.3% [−8.3;−4.2] (from 29.0% [27.0;30.9] to 27.1% [25.2;29.1]) observed in girls (Table 2). In CON all the observed changes were independent of gender (data not shown).

**Table 2:** Relative changes (%) from baseline (with 95%CI) in boys and girls in the intervention school.

	Intervention school				
Outcome	Boys		Girls		*p*-value
	Relative change (%)	*n*	Relative change (%)	*n*	
YYIR1C	30 [22;39]***	77	32 [17;47]***	73	0.88
AAT	−2.8 [−3.3;−0.3]*	74	−2.4 [−3.9;−0.9]**	71	0.57
Weight	2.8 [1.9;3.6]***	75	2.7 [2.0;3.5]***	77	0.73
Total muscle mass	8.2 [7.0;9.5]***	75	6.1 [4.9;7.4]***	77	<0.05
Total fat percentage	−12.8 [−15.5;−10.2]***	75	−6.3 [−8.3;−4.2]***	77	<0.001
MAP	−3.8 [−6.0;−1.6]***	70	−2.2 [−4.4;−0.1]*	74	0.24

YYIR1C, Yo-Yo intermittent recovery 1 children’s test; AAT, arrowhead agility test; MAP, mean arterial pressure. The *p* value of the unpaired *t*-test comparing the relative changes between genders is presented. *, **, *** Denotes significant within-gender change from baseline at *p* < 0.05, *p* < 0.01, and *p* < 0.001.

### 3.5 Sub-group analysis – the effect of age-group

The intervention-induced changes in CRF and agility were similar between age-groups (Table 3). However, the increase in body weight and muscle mass was statistically most pronounced in grade 4–6 (level II), while the reduction in fat mass was statistically independent of age-group (Table 3). Finally, a significant between-group effect was observed for MAP, with the pupils in grade 1-3 (level) and 7–9 (level III) demonstrating significant reductions in MAP while no effect was observed in the pupils in grade 4–6 (level II).

**Table 3:** Relative changes (%) from baseline (with 95%CI) in different age-groups in the intervention school.

	Intervention school						
Outcome	Level I		Level II		Level III		*p*-value
	Relative change (%)	*n*	Relative change (%)	*n*	Relative change (%)	*n*	
YYIR1C	32 [8;56]***	60	27 [16;39]***	54	41 [30;51]***	36	0.58
AAT	−1.6 [−3.1;−0.1]*	60	−3.0 [−4.9;−1.2]**	50	−2.2 [−4.5;0.2]	35	0.29
Weight	2.2 [0.9;3.5] **	54	3.8 [2.9;4.9]***	52	2.1 [1.3;2.9]***	46	0.01
Total muscle mass	7.2 [5.0;9.3]***	54	8.6 [7.1;10.0]***	52	6.0 [4.8;7.1]***	46	0.01
Total fat percentage	−8.7 [−11.5;−5.9]***	54	−10.3 [−13.1;−7.6]***	52	−8.1 [−11.2;−5.0]***	46	0.63
MAP	−3.3 [−5.9;−0.7]*	51	−0.6 [−3.2;2.0]	51	−4.9 [−7.6;−2.3]***	42	0.01

YYIR1C, Yo-Yo intermittent recovery 1 children’s test; AAT, arrowhead agility test; MAP, mean arterial pressure. The *p* value of the one-way ANOVA comparing the relative changes between levels is presented. *, **, *** Denotes significant within-age-group change from baseline at *p* < 0.05, *p* < 0.01, and *p* < 0.001.

## 4 Discussion

The present study investigated the impact of a 10-week implementation of the FIT FIRST FOR ALL concept on physical performance and general health status across an entire school, encompassing pupils aged 7–16 years. Notably, the 10-week implementation of the FIT FIRST concept markedly improved cardiorespiratory fitness, agility, and body composition, while no statistical between-group effect existed for jump performance, postural balance, blood pressure and resting heart rate. The beneficial health impact of the FIT FIRST physical activity sessions was partly affected by gender and age, but in general physical health status was improved in both boys and girls, as well as across all age groups.

Cardiorespiratory fitness was evaluated using the YYIR1C, a validated and reliable test for determining endurance capacity in children (33). In the present study, we observed a 31% increase in YYIR1C performance in the intervention school, whereas no statistical changes existed in the control school. In accordance with our findings, other studies utilizing intense training modalities in school settings over a comparable time-period have reported similar improvements in cardiorespiratory fitness levels (39) and the other cardiovascular health markers (15, 22, 40). Moreover, a study comparing high-intensity interval training with moderate-intensity interval training in 10–13-year-old boys in a real-life school environment reported superior improvements in maximum oxygen uptake in favor of the high-intensity interval training group (6.1 mL/kg/min vs. 3.8 mL/kg/min) (41). The high intensity of the present study is confirmed by heart rate measurements during the FIT FIRST sessions (data not shown), which revealed a high cardiovascular load according to the American College of Sports Medicine (ACSM) guidelines for heart rate intensity zones (42). Taken together, the 10-week FIT FIRST intervention significantly improved cardiorespiratory fitness in children aged 7–16 years when implemented across an entire school. These findings hold clinical relevance, as meta-analysis data confirm an association between childhood cardiorespiratory fitness and adult health status (43). Furthermore, a cohort study tracking 748 school children over 34 years demonstrated that childhood cardiorespiratory fitness explained 30 and 16% of the variance in young- and mid-adulthood cardiorespiratory fitness (44). Therefore, early interventions targeting cardiorespiratory fitness may play a crucial role for promoting health in adulthood.

In relation to agility, which is considered an important factor for physical, mental and social development in children (45), we observed a ~ 2% increase in AAT scores in the intervention school, whereas a ~ 3% decrease was observed in the control school. These findings are supported by other studies in children (46, 47). Given the important role of agility as a proxy for proficiency across different sports and types of physical challenges, it merits classification as a key component for developing motor skill proficiency. This, in turn, significantly enhances the probability of increased participation in sports and physical activity (48, 49). In contrast to agility, no between-group effect was observed for changes in standing long jump and balance. Improvements in jump and balance have been observed in other studies with longer interventions (22), whereas studies using comparable intervention durations to the present study have failed to identify significant improvements (40).

Childhood obesity, a pervasive global health concern, poses significant risks for the development of various lifestyle-related diseases later in life (50). For example, in 2016, 124 million children and adolescents worldwide aged 5–19 years were estimated to suffer from obesity and 213 million were deemed as overweight (51). Within the current cohort, 40% of all pupils were identified as overweight or obese based on calculated BMIz scores (≥ 1 SD) (29). However, the implementation of the 10-week FIT FIRST training protocol yielded notable improvements in body composition. Specifically, children in the intervention school experienced a 2.3% reduction in body fat content and a nearly 1.5 kg increase in muscle mass. Previous research within our research group demonstrated improvements in bone health among 8–10-year-old children following FIT FIRST training, albeit without significant changes to body composition, thus contrasting with the present findings (22). Conversely, other school-based physical activity interventions, such as the 11 for Health intervention program targeting 10–13-year-old girls and boys in different cultural settings, have demonstrated improvements in body composition (15, 22, 40). Additionally, a recent study by Cao et al. (41) exposed obese 10–13-year-old boys to high-intensity interval training three times weekly for 12 weeks in a school setting, which resulted in similar reductions in body fat content as in the present study. Furthermore, Cao and colleagues reported concomitant positive effects on visceral adipose tissue and low-density lipoprotein cholesterol (41), highlighting the efficacy of frequent high intensity interval training in enhancing cardiometabolic health. A notable aspect of our study is the involvement of an entire school in the high-intensity training intervention, yielding clinically relevant improvements in body composition in both genders and across all age-groups. However, it is imperative to note that the measurement of muscle mass and body fat content using present bioimpedance technology presents a limitation. Recent evidence suggests that while the InBody device exhibits high inter-device reliability, it may underestimate muscle mass and overestimate body fat percentage in 10–12-year-old-children (27), necessitating cautious interpretation of the body composition values. The strength of using this methodology is that it is affordable and time efficient and allows measurements in a school setting to be carried out for all pupils supplying quantitative date also in regions far removed from advanced state-of the art methods such as DXA scans (52, 53).

No statistical between-group effect was observed for changes in arterial blood pressure and resting heart rate in the present study. This contrasts with other school-based exercise interventions such as the 11 for Health (15, 40) despite the higher training volume in the present study. Across several of our school-based interventions, a reduction in systolic blood pressure of ~5 mmHg has been observed (15, 40, 54), which in adults corresponds to a 13% reduction in cardiovascular mortality and morbidity (55). While a direct risk assessment in children is not feasible, if such a response can persist into adulthood, it could yield significant cardiovascular health benefits.

The primary aim of the present study was to evaluate the impact of the FIT FIRST concept on physical health outcomes at the school-level. Upon assessing potential gender differences in the relative changes (%) from baseline in subgroup analysis, similar improvements were observed in both genders across most variables. However, some disparities emerged between boys and girls in body composition changes Specifically, muscle mass increased more in boys (8.2%) compared to girls (6.1%). Similarly, the reduction in body fat content from 22.5 to 19.6% in boys (a ~ 13% reduction), was significantly larger than the decline from 29.0 to 27.1% in girls (a ~ 6% reduction). These discrepancies may, in part, be attributed to biological maturation, with boys, particularly those undergoing puberty, experiencing a more pronounced increase in muscle mass (56, 57). Maturation assessments were not conducted during the study. However, the boys (2.0% increase) and the girls (2.4% increase) in the control school displayed a statistically similar (*p* = 0.65) (data not shown) increase in muscle mass over the 10-week intervention period, which indicates that the gender-specific adaptations in body composition observed in the intervention school are a direct effect of the intervention. Moreover, with few exceptions, consistent improvement patterns were observed in children across all age-groups. However, the increase in body weight and muscle mass was most pronounced in level II. The most notable between-age-group effect was observed for MAP, with the oldest children in level III displaying the most significant effect. This finding could be attributed to their numerically higher pre-test values. Collectively, our findings demonstrate several health-beneficial effects from participating in the FIT FIRST FOR ALL school-based intervention. Particularly the marked improvements in cardiorespiratory fitness and body composition, which are widely accepted markers for overall health (55).

Although three FIT FIRST sessions were planned weekly for 10 weeks, the actual completion rate was 2.1–2.3 sessions across all age-groups, and pupil attendance averaged 82% (range 75–87%). Despite this relatively modest training volume, substantial health-beneficial effects were observed, suggesting that the efficacy of high intensity training (58) also applies to children. The level of completion compared to the planned three-time weekly program, and the level of attendance of pupils, shows an important factor when planning school interventions in realistic settings. Especially when an intervention is carried out on a school-wide basis with all classes in school attending the program. If schools are to be an arena, where we wish to promote physical activity programs to reach an intended volume of physical exercise, we must consider the realistic completion rate. This is an important outcome of the present study, in relation to promoting political understanding and acceptance of increased time for physical activity during school time.

It is a strength that the two schools involved in the present study are comparable. Both schools have the same size and are in the south and north end of an island with around 4.500 inhabitants. The two main towns of the islands, where the two schools are, are culturally and socioeconomically equal. It is also a strength that the entire school participated in the intervention, rather than targeting specific age-groups, enhancing the generalizability of our findings. Additionally, all physical activity sessions were supervised by trained PE-teachers following standardized manuals, ensuring consistency and quality in each FIT FIRST session. However, a limitation of the study is the non-randomized selection of the intervention and control school, which was caused by necessary logistics. Finally, it should be noted that the sample size may have been too low in terms of differentiating the gender- and age-group effects, and hence the comparisons between genders and age-groups should be interpreted with caution due to potential statistical type II errors.

## 5 Conclusion

In conclusion, our findings verify the main hypothesis that the FIT FIRST FOR ALL concept causes beneficial health effects in cardiorespiratory fitness and body composition in an entire school over a 10-week period in children having a age range from 7 to 16 years. Regarding the secondary endpoints, the concept also improved agility at school level, but in contrast to our hypothesis failed to impact balance, countermovement jump performance, resting heart rate or blood pressure. In relation to our explorative hypothesis cardiorespiratory fitness was augmented independent of gender and age group, while change scores in muscle mass and body fat content appeared to improve more in boys than in girls.

## Data availability statement

The datasets discussed in this article are not readily available due to the vulnerability of the data, which was collected in a small-scale society and could lead to recognition of the subjects. While the research team is committed to full transparency and openness, the protection of the participants requires that data sharing be strictly controlled. Requests to access the datasets should be directed to the corresponding author.

## Ethics statement

The studies involving humans were approved by the Research Ethics Council of the Faroe Islands. The studies were conducted in accordance with the local legislation and institutional requirements. Written informed consent for participation in this study was provided by the participants’ legal guardians/next of kin.

## Author contributions

HO: Writing – review & editing, Writing – original draft, Visualization, Validation, Supervision, Software, Resources, Project administration, Methodology, Investigation, Funding acquisition, Formal analysis, Data curation, Conceptualization. TS: Writing – review & editing, Writing – original draft, Visualization, Validation, Software, Methodology, Investigation, Formal analysis, Data curation, Conceptualization. BD: Writing – review & editing, Writing – original draft, Validation, Supervision, Resources, Project administration, Methodology, Investigation, Conceptualization. PK: Writing – review & editing, Writing – original draft, Validation, Supervision, Resources, Project administration, Methodology, Funding acquisition, Conceptualization. ML: Writing – review & editing, Writing – original draft, Validation, Supervision, Resources, Project administration, Methodology, Investigation, Funding acquisition, Formal analysis, Data curation, Conceptualization. M-BS: Writing – review & editing, Writing – original draft, Validation, Supervision, Resources, Project administration, Methodology, Investigation, Conceptualization. MM: Writing – review & editing, Writing – original draft, Validation, Supervision, Resources, Project administration, Methodology, Investigation, Funding acquisition, Conceptualization.
